# Mitochondrial sequence data expose the putative cosmopolitan polychaete *Scoloplos armiger *(Annelida, Orbiniidae) as a species complex

**DOI:** 10.1186/1471-2148-6-47

**Published:** 2006-06-15

**Authors:** Christoph Bleidorn, Inken Kruse, Sylvia Albrecht, Thomas Bartolomaeus

**Affiliations:** 1 Animal Systematics and Evolution, Institute for Biology, Zoology, Free University Berlin, Koenigin-Luise-Str. 1-3, D-14195 Berlin, Germany; 2 Unit of Evolutionary Biology/Systematic Zoology, Institute of Biochemistry and Biology, University of Potsdam, Karl-Liebknecht-Strasse 24-25, Haus 26, D-14476 Potsdam-Golm, Germany; 3 Smithsonian Marine Station at Fort Pierce, 701 Seaway Drive, Fort Pierce, Florida, USA; 4 Institute for Zoo and Wildlife Research, 10252 Berlin, Germany

## Abstract

**Background:**

Polychaetes assigned as *Scoloplos armiger *(Orbiniidae) show a cosmopolitan distribution and have been encountered in all zoogeographic regions. Sibling *S. armiger*-like species have been revealed by recent studies using RAPDs and AFLP genetic data. We sequenced a ~12 kb fragment of the *Scoloplos *cf. *armiger *mitochondrial genome and developed primers for variable regions including the 3' end of the *cox3 *gene, *trnQ*, and most of *nad6*. A phylogenetic analysis of this 528-nucleotide fragment was carried out for *S. armiger*-like individuals from the Eastern North Atlantic as well as Pacific regions. The aim of this study is to test the cosmopolitan status, as well as to clarify the systematics of this species complex in the Eastern North Atlantic, while using a few specimens from the Pacific Ocean for comparision.

**Results:**

Phylogenetic analysis of the *cox3*-*trnQ*-*nad6 *data set recovered five different clades of *Scoloplos *cf. *armiger*. The fragment of the mitochondrial genome of *Scoloplos *cf. *armiger *is 12,042 bp long and contains 13 protein coding genes, 15 of the 22 expected tRNAs, and the large ribosomal subunit (*rrnl*).

**Conclusion:**

The sequenced *cox3-trnQ-nad6 *fragment proved to be very useful in phylogenetic analyses of *Scoloplos *cf. *armiger*. Due to its larger sampling scale this study goes beyond previous analyses which used RAPD and AFLP markers. The results of this study clearly supports that *Scoloplos armiger *represents a species complex and not a cosmopolitan species. We find at least two *S. armiger*-like species within the Pacific region and three different *S. armiger*-like species in the North Atlantic. Implications for the taxonomy and the impact on ecological studies are discussed.

## Background

Polychaetes assigned as *Scoloplos armiger *are common as dominant species in ecological surveys in different marine habitats. Benthic surveys have shown that *S. armiger *represents one of the dominant macrofauna species in a Norwegian fjord [1], in a Portuguese estuary [2], and in the Peter the Great Bay in the Sea of Japan [3]. Besides its wide ranging distribution, *S. armiger *also plays a more or less important role in recent ecological studies. It has been identified as a possible intermediate host for the flatfish nematode *Cucullanus heterochrous *[4] and the population dynamics of *S. armiger *and its predator *Nephtys hombergii *(Nephtyidae) on intertidal flats in the Netherlands' part of the Wadden Sea are well studied [5].

*Scoloplos armiger *(Orbiniidae) has been reported to show a cosmopolitan distribution and has been encountered in all zoogeographic regions [6,7] where it is present from the intertidal to the subtidal [8,9]. In the North Sea region it is one of the most common polychaetes and a direct development in egg cocoons was observed at many intertidal flats [8]. In additional to these well known and eye-catching cocoons, free swimming pelagic larvae of these worms have been reported from the North Sea near the island of Helgoland, Germany [10].

For many marine invertebrate species a worldwide distribution has been reported. At least four hypotheses can reasonably explain such a distribution pattern; (a) truly cosmopolitan species, (b) cosmopolitan morphospecies which correspond to genetically distinct species [11,12], (c) poor taxonomic understanding of a taxon, causing "the cosmopolitan syndrome" [13-15] and (d) cosmopolitans where the current range distribution is the result of human introductions. An example for the latter is the reef-building serpulid *Ficopomatus enigmaticus *which can be found in brackish waters of warm-temperate regions all over the world, and which is supposed to be distributed through human shipping [16]. However, most reports of cosmopolitan distributional ranges of marine invertebrate species after application of molecular methods turned out to be the result of an over-conservative taxonomy [17-19].

Among marine invertebrates, polychaete annelids have a high frequency of cosmopolitan species [20,21]. Polychaetes like *Owenia fusiformis *(Oweniidae), *Sternaspis scutata *(Sternaspidae) and *Scoloplos armiger *(Orbiniidae) are recorded from all oceans in different depths and nearly all temperate regions [7,15,22]. However *O. fusiformis *later has been found to consist of more than one species [23].

Only a few genetic studies investigated such "cosmopolitan" polychaetes and most of them did not use discrete nucleotide data. RAPDs and ITS sequence data confirmed the amphi-Atlantic distribution pattern of the ctenodrillid *Ctenodrilus serratus *[24]. The worldwide distributed *Petitia amphophthalma *(Syllidae) has been investigated with RAPD markers [25,26] which do not support the cosmopolitan status of this taxon. The phylogeography of the invasive sabellid *Sabella spallanzanii *was investigated using nuclear markers [27] and human introduction to Australia due to ballast water has been suggested for this polychaete. The cosmopolitan status of *Hesionides areneria *(Hesionidae) was confirmed using RAPD markers [28], nevertheless it cannot be ruled out that lack of differences in the band pattern of RAPDs is due to primer choice. In contrast to this, the cosmopolitan status of another hesionid (*Hesionides gohari*) was not supported by RAPD data [29].

Interestingly, none of these studies used mitochondrial markers which are commonly used for phylogeographic studies in other animal groups [30]. This might be due to the lack of suitable primers for the amplification of variable regions of the mitochondrial genome. Although many polychaetes are recorded from different zoogeographic regions, truly cosmopolitan species seem to be rare, and in many cases taxonomy is unable to distinguish between morphologically similar taxa [31]. It is supposed that widely distributed species are frequently being found to consist of distinguishable subspecies or siblings when examined in sufficient detail [12].

In a series of papers it has been shown with RAPD data, AFLPs, cross-breeding experiments and investigation of the sperm morphology, that different developmental traits of *Scoloplos armiger *collected near the island of Sylt (Germany) belong to two distinct *Scoloplos *species [32-34]. This means that two sympatric sibling species of *Scoloplos *cf.* armiger *occur in the North Sea: one living in the intertidal with egg cocoons and one living subtidally with pelagic larvae.

**Figure 1:**
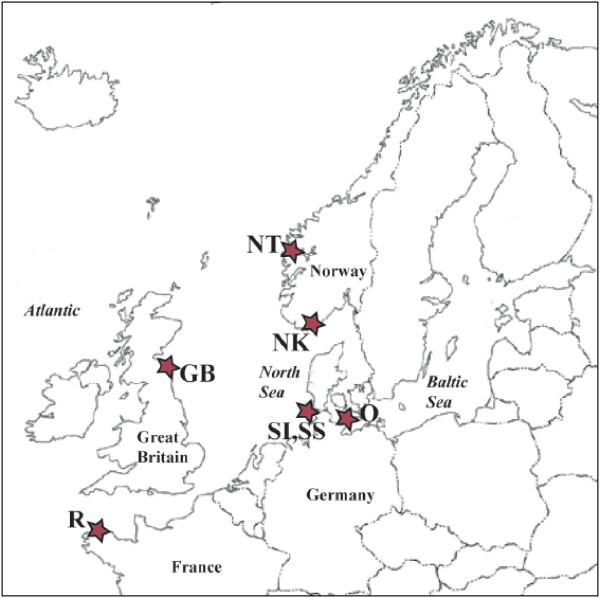
**Map of collection sites in Europe**. Abbreviations are according to the labeling of individuals as given in Table 1: NT, Trondheimsfjord (Norway); NK, Kristiansand (Norway); GB, Low Newton by the Sea, (Great Britain); R, Roscoff (France); SI, Sylt intertidal (Germany); SS, Sylt subtidal (Germany); O, Fehrmanns Belt (Germany).

The aim of the present study is to investigate the status of different *Scoloplos *cf. *armiger *populations in the Northern East Atlantic (see Fig. 1 for collection sites) and the Northern East Pacific using mitochondrial markers. For this purpose we sequenced a 12 kb fragment of the mitochondrial genome of *Scoloplos *cf. *armiger *to develop primers for a variable mitochondrial region. Our present study gives no support for a cosmopolitan distribution of *Scoloplos *cf. *armiger *and phylogenetic analyses of the investigated populations reveal five distinct reciprocal monophyletic clades of *Scoloplos *cf. *armiger*.

## Results

### Genome organisation, base composition, and codon usage of the mitochondrial genome of *Scoloplos *cf. *armiger*

**Figure 2:**
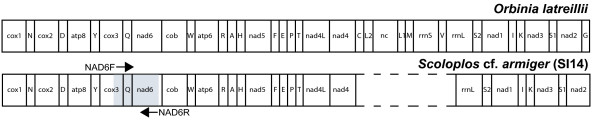
**Mt genomic features**. Gene arrangements of the mitochondrial genomes of *Scoloplos *cf. *armiger *(above) and *Orbinia latreillii *(below). Primer sites for the *cox3*-*trnQ*-*nad6 *fragment marked by arrows.

The fragment of the mitochondrial genome of *Scoloplos *cf. *armiger *individual SI14 is 12,042 bp long and contains 13 protein coding genes, 16 of the 22 expected tRNAs, and the large ribosomal subunit (*rrnl*). As in the case for all annelids so far studied all genes are transcribed from the same strand. One difference found in the gene arrangement of *Scoloplos *cf. *armiger *when compared with the other known orbiniid mitochondrial genome of *Orbinia latreillii *[35] is that the gene *trnG *is missing within the so far sequenced portion of *Scoloplos *cf. *armiger *(Fig. 2). The mitochondrial genome is AT-rich (63.66%), and the base frequencies are A = 0.31, C = 0.24, G = 0.12, and T = 0.33.

All 13 protein-coding genes typically found in metazoan mtDNA [36] are identified for *Scoloplos *cf. *armiger*. In 12 of these AUG is used as a start codon. The exception is *cox3*, for which sequence alignment comparison with other annelids reveals the use of GUU as alternative start codon. An alternative start codon is also found for the same gene in *Orbinia *[35]. Except for *nad1 *complete stop codons (eight times UAA and three times UAG) are found in all other protein coding genes in *Scoloplos *cf. *armiger *(with the exception that the 3' end of *nad4 *is not completely sequenced).

### Phylogenetic analysis of the *cox3 – trnQ – nad6 *data set

Within 55 individuals we found 25 unique sequence haplotypes for which we produced an alignment spanning 528 characters. Of these 528 characters, 245 characters are constant, 105 characters are variable but parsimony uninformative, and 178 are parsimony informative. The nucleotide composition is AT biased, as is it common for polychaete mitochondria [35,37] and the empirical base frequencies are A = 0.318, C = 0.227, G = 0.113, and T = 0.342. The chi-square test of homogeneity of base frequencies across taxa resulted in no significant *P*-values (chi-square = 25.28, df = 72, *P *= 0.999).

**Figure 3:**
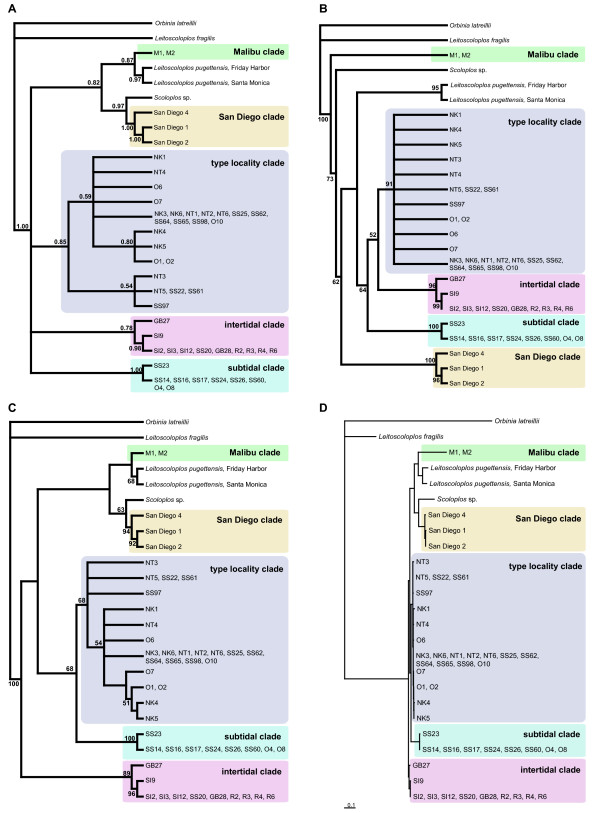
**Phylogenetic relationships of *Scoloplos***. (A-C) Topologies generated by different phylogenetic analyses: (A) Majority-rule consensus of the Bayesian analysis (GTR+I+Γ) with posterior probabilities at the nodes; (B) Strict consensus of the 40 equally parsimonious trees generated by maximum parsimony (MP) analyses with bootstrap values at the nodes; (C) Maximum Likelihood topology (GTR+I+Γ) with bootstrap values at the node; (D) Maximum Likelihood tree with branch lengths.

The application of the different phylogenetic methods yielded different tree topologies (Fig. 3), but the same major clades are recovered by all. The MP approach yielded 40 equally parsimonious trees (each with 593 steps) which are presented as a strict consensus tree (Fig. 3b). Five different reciprocal monophyletic clades of *Scoloplos *cf. *armiger *are recovered: a clade containing the individuals from Malibu ('Malibu clade'), one containing the individuals from San Diego ('San Diego clade'), one containing the intertidal specimens from Roscoff, Low Newton by the Sea, and Sylt ('intertidal clade'), one containing individuals from Sylt and Fehrmanns Belt which were collected from the subtidal ('subtidal clade'), and one also containing individuals from the latter two locations, as well as individuals from Trondheim and Kristiansand. The sample site in Kristiansand is located near the type locality and so this clade is named the 'type locality clade'. The 'Malibu clade' is represented by two identical sequences and the monophyly of the other clades is well supported through bootstrap values and Bayesian posterior probabilities (BPP) (Fig. 3). The relationship between these clades remains unclear, but no analysis recovered a monophyletic *Scoloplos *cf. *armiger *clade. ML and Bayesian inference indicates that the 'Malibu clade' is closely related to *Leitoscoloplos pugettensis*, but this relationship is only poorly supported through BPP (0.87), as well as that there is a sistergroup relationship between the 'San Diego clade' and *Scoloplos *sp. The relationships between the three clades from European waters are also not clear. Whereas MP recovers a sister group relationship between the 'type locality clade' and the 'intertidal clade' (Fig. 3b), a sister group relationship between the 'subtidal clade' and the 'type locality clade' is suggested by the most likely tree (Fig. 3c, 3d). The majority rule tree of the Bayesian inference does not resolve this issue (Fig. 3a).

As expected from this phylogenetic analyses, comparison of average nucleotide diversity between different *Scoloplos *cf. *armiger *clades shows that variation between clades (Table 2) are much higher than within clades (Table 3).

**Table 1:** Sampling sites, sequenced individuals, and GenBank accession numbers of analysed taxa.

Taxon	Location	Individuals	Accession-Nr.
*Orbinia latreillii*	Roscoff, France		AY961084
*Leitoscoloplos fragilis*	Little Buttermilk Bay, MA, USA		DQ408432
*Leitoscoloplos pugettensis*	Friday Harbor, WA, USA		DQ408433
*Leitoscoloplos pugettensis*	Santa Monica, CA, USA		DQ408434
*Scoloplos *sp.	Morro Bay, CA, USA		DQ408435
*Scoloplos *cf. *armiger*	Malibu Beach (CA, USA), intertidal	M1, M2	DQ408436–DQ408437
*Scoloplos *cf. *armiger*	San Diego (CA, USA), subtidal	SASD1, SASD2, SASD4	DQ408438–DQ408440
*Scoloplos *cf. *armiger*	Buholmsanden, Kristiansand (Norway)	NK1, NK3, NK4, NK5, NK6	DQ408441–DQ408445
*Scoloplos *cf. *armiger*	Sletvik, Agdenes, Trondheimsfjord, (Norway)	NT1, NT2, NT3, NT4, NT5, NT6	DQ408446–DQ408451
*Scoloplos *cf. *armiger*	Fehrmanns Belt, Baltic Sea (Germany), subtidal	O1, O2, O4, O6, O7, O8, O10	DQ408477–DQ408484
*Scoloplos *cf. *armiger*	Sylt (Germany), intertidal	SI2, SI3, SI9, SI12	DQ408452–DQ408455
*Scoloplos *cf. *armiger*	Sylt (Germany), subtidal	SS14, SS16, SS17, SS20, SS22, SS23, SS24, SS25, SS26 SS60, SS61, SS62, S64, SS65, SS97, SS98	DQ408456–DQ408470
*Scoloplos *cf. *armiger*	Roscoff (France), intertidal	R2, R3, R4, R6	DQ408473–DQ408476
*Scoloplos *cf. *armiger*	Low Newton by the Sea (Great Britain), intertidal	GB27, GB28	DQ408471–DQ408472

**Table 2:** Average Kimura Two-Parameter distances calculated from Transition and Transversion changes between different *Scoloplos *cf. *armiger *clades

	Malibu	San Diego	type locality	Sylt intertidal
San Diego	0.238			
type locality	0.209	0.132		
Sylt intertidal	0.237	0.136	0.053	
Sylt subtidal	0.231	0.149	0.088	0.095

**Table 3:** Average Kimura Two-Parameter distances calculated from Transition and Transversion changes within different *Scoloplos *cf.* armiger *clades

Malibu	not calculated
	
San Diego	0.009
type locality	0.008
Sylt intertidal	0.013
Sylt subtidal	0.002

**Figure 4:**
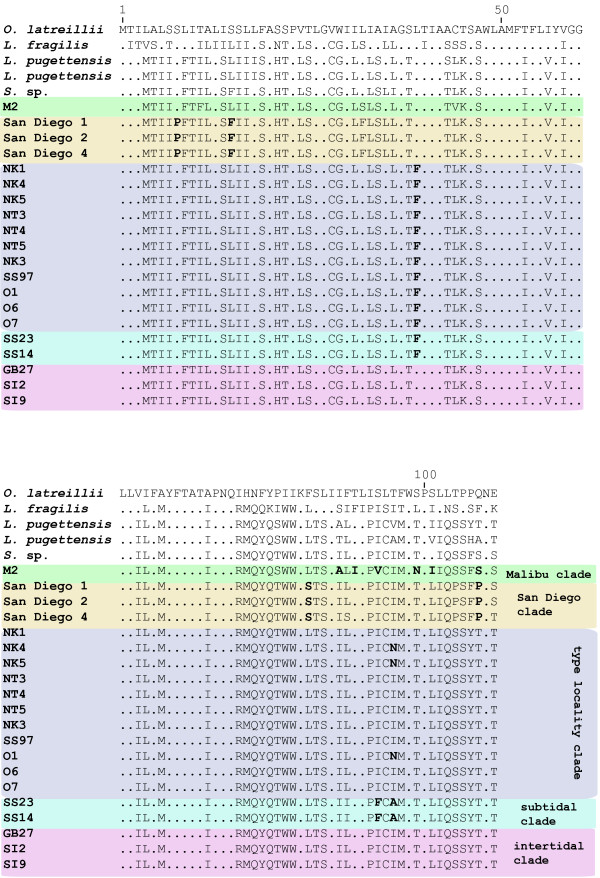
**Amino acid alignment of the *nad6 *gene**. Differences within the *Scoloplos *cf. *armiger *group marked in bold.

There are several amino acid changes within the *nad6 *gene (Fig. 4). Within the *Scoloplos *cf. *armiger *group unique amino acid substitutions are present for the 'Malibu clade', the 'San Diego clade', the 'subtidal clade', and the 'type locality clade'.

### *trnQ *secondary structures

**Figure 5:**
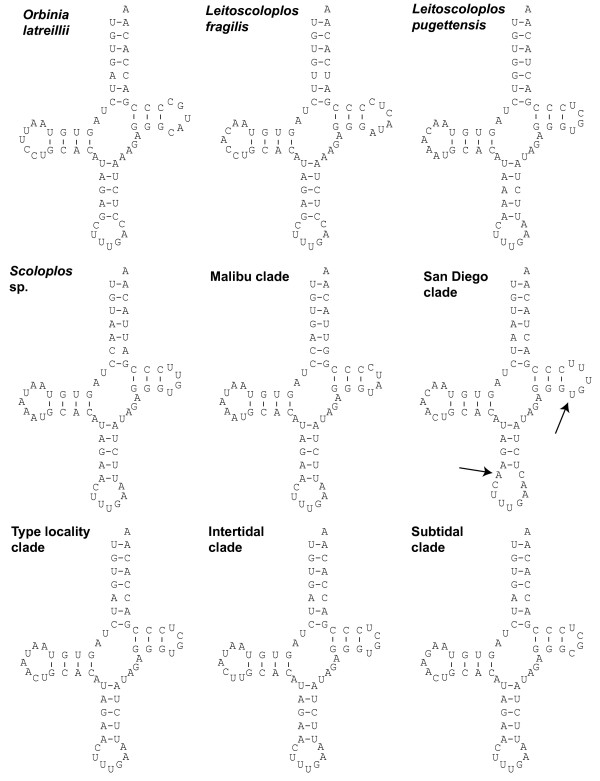
**Proposed secondary structure of the *trnQ *gene of different *Scoloplos *cf. *armiger *clades**. There are no different secondary structures within the clades and as such only secondary structure of *trnQ *one representative of each clade is shown. Discussed feature are marked with an arrow.

Proposed *trnQ *secondary structures for all clades/taxa are given in Figure 5 and all possess the common cloverleaf structure with an acceptor stem, TΨC stem and loop, anticodon stem and loop, and DHU stem and loop (clockwise in Fig. 5). Secondary structures are identical within each clade/taxon and therefore only structures of one individual are shown. The secondary structure predicted for the 'San Diego clade' differs from the other *Scoloplos *cf. *armiger *taxa in possessing 5 bp instead of 4 bp in the TΨC loop, 4 paired bases instead of 5 paired bases in the anticodon stem, and 9 bp instead of 7 bp in the anticodon loop.

## Discussion

### Relationships of different *Scoloplos *cf. *armiger *populations

The results of the phylogenetic analyses of the *cox3-trnQ-nad6 *data set clearly supports that *Scoloplos armiger *represents a species complex and not a cosmopolitan species. We find at least two *S. armiger*-like genetic clades within the Pacific region and these are more closely related to other Pacific species (*Leitoscoloplos pugettensis*, *Scoloplos *sp. from Morro Bay, CA) than to the European *Scoloplos *cf. *armiger *clades. Under the phylogenetic species concept sensu Mishler and Theriot [38] it is parsimonious to assume that these clades represent distinct evolutionary lineages which should be considered as species distantly related to *S. armiger*. We identified three well supported reciprocal monophyletic clades within European *Scoloplos *cf.* armiger*. Applying the phylogenetic species concept sensu Mishler and Theriot [38] to our data, we hypothesize the presence of at least three different species (subtidal clade, type locality clade, intertidal clade) formerly referred to as *S. armiger *in the North Atlantic.

The inference of a monophyletic clade containing all intertidally collected interviduals from European waters confirms the results that individuals that are producing egg-cocoons and live on intertidal flats represent a distinct evolutionary lineage rather than part of a *S. armiger *species with different developmental modes [32]. Surprisingly the results show that in subtidal areas of the North Sea and Baltic Sea there are two clearly separated genetic clades of *Scoloplos *cf.*armiger*, which are also distinct in amino acid data: the 'subtidal clade' and the 'type locality clade'. Whereas we found both genetic types in the North Sea and Baltic Sea samples in sympatry, only one of these clades seems to be present in the Norwegian samples, which include the type locality. As pointed out before, we consider it likely that these two genetic clades represent two different species. However, at this point the possibility must be considered that processes unrelated to speciation have generated reciprocal monophyletic mtDNA haplotype lineages [39], especially for the separation of the 'subtidal clade' and the 'type locality clade'. This hypothesis should be tested with additional data, e.g. by application of independent nuclear markers.

Differences in sperm morphology and in the length of anal cirri of benthic juveniles between intertidal and subtidal populations have been reported by Kruse & Reise [33], but they did not find any such differences or variation within the 'subtidal clade'. The same holds true for chaetal characters. Furcate chaetae are present in abdominal segments of individuals of the subtidal populations, but these are lacking in intertidal individuals [40]. These characters were also compared with individuals from the type locality (Kristiansand, Norway), but no differences to the subtidal individuals from the Sylt population are found. However, our results explain why significantly higher genetic diversity has been found in a RAPD study within the subtidal populations [32]. It is very likely that this has been caused by mixing of two cryptic species which together contribute to an ostensible high variability.

The question emerges if there are ecological differences between the two cryptic subtidal species. Study of the ecological background of the two *Scoloplos *cf. *armiger *species in the Wadden Sea (North Sea, Germany) has revealed that there is a higher tolerance against sulphide and hypoxia for intertidal individuals, which can be interpreted as an adaptation to intertidal habitats being cut off from supply with oxygenated sea water during low tide. However, no unusual high variation of the physiological tolerance of subtidal *Scoloplos *cf.* armiger *individuals is revealed by these physiological studies [34].

Interestingly, it can be observed that intertidal and subtidal populations both spawn their egg cocoons and pelagic larvae respectively in spring and additionally pelagic larvae from subtidal populations were present in autumn. Spawning asynchrony is typical for marine sibling species living in sympatry [12,41] and in the future it needs to be tested if this is realized in the two clades with subtidal *Scoloplos *cf.* armiger*: one spawning in autumn and one in spring.

### Implications for *Scoloplos *taxonomy

It is obvious from this analysis, as well as from molecular study of phylogenetic relationships of Orbiniidae [42], that the genus *Scoloplos *is not monophyletic. Characters currently used for genus diagnoses in orbiniids are highly variable within this group and are not suitable for cladistic analysis [42]. The status of the worldwide distributed *Scoloplos armiger *was doubted by some authors before [43]. *S. armiger *is a species with variable morphological characters. Descriptions of this species differ so widely that more than one species may have been confused [44].

From the present analysis it becomes clear that at least three additional *Scoloplos *species should be erected within the species complex currently referred to as *Scoloplos armiger*: the Malibu clade, San Diego clade, and the intertidal clade. Additional to this, according to our data the existence of sibling species within the subtidal populations of the Eastern North Atlantic is highly likely (type locality clade, subtidal clade).

Whereas the descriptions for the Malibu clade and the intertidal clade are in preparation, the two other clades need further investigation before formal description. In future, the name *Scoloplos armiger *should be restricted to the type locality clade.

With the present molecular analysis at hand it is very likely that different species have been mixed in previous ecological studies. Whereas it seems reasonable that European *Scoloplos armiger*-like individuals from tidal flats can be assigned to the intertidal clade, the status of subtidal populations remains more ambiguous. In the future reports of *S. armiger *from non-European waters should be treated with caution. The analysis of the few included pacific individuals clearly indicates that these represent different species, which appear to be distantly related to European *Scoloplos species*. It would be interesting to include Mediterranean as well as subtidal species from the Sea of Japan, White Sea, and British waters in future studies to clarify the species status of different *S. armiger*-like populations and to understand their distribution.

### *Scoloplos *mitochondrial genome data

This is the first attempt to use mitochondrial data to distinguish between *Scoloplos *species which has proven to be very powerful. We present the first nearly complete mitochondrial genome (ca. 12 kb) for this genus including all protein coding genes. Long-PCR's ranging from *nad4 *to *16S *were not successful. Problems with amplifying the part of the mitochondrial genome including the putative control region have also been reported by others [37]. Compared with the mitochondrial genome of the orbiniid *Orbinia latreillii *[35] two translocations of tRNA genes must be assumed. This shows that gene rearrangements might be more frequent in annelids than previously assumed [35,37].

We analysed a fragment of the mitochondrial genome starting from the 3'-end of *cox3*, continuing over the complete *trnQ*, and finishing after a large part of *nad6*. Analysis of the secondary structure of the *trnQ *genes in our data set show the typical functional cloverleaf structure, which indicates that we most likely did not encounter mitochondrial pseudogenes, so called numts [45]. The sequenced fragment in this study proved to be very useful in phylogenetic analyses for the distinction of different clades. Due to its larger sampling scale this study goes beyond previous analyses which used RAPD and AFLP markers [32].

## Conclusion

The phylogenetic analysis of mitochondrial sequence data (*cox3-trnQ-nad6 *fragment) reported here revealed that *Scoloplos armiger *represents a species complex and not a cosmopolitan species. We find at least two *S. armiger*-like species within the Pacific region and two or three different *S. armiger*-like species in the North Atlantic. One of these species is represented by the intertidal clade, for which previous studies clearly had supported species status. Further morphological as well as genetical investigations of *S. armiger*-like individuals from the subtidal and the type locality clade will shed additional light on a cryptic speciation within *Scoloplos*. It appears likely that inclusion of more *Scoloplos *cf.* armiger *specimens from different parts of the world would add more species to this complex.

## Methods

### Samples, identification, and DNA extraction

Individuals of *Scoloplos *cf. *armiger *and other orbiniids were collected at different sample locations (Table 1, see Fig. 1 for collection sites of the European *Scoloplos *samples) and preserved in 99% ethanol. Pacific *Scoloplos *species of the Malibu clade have been collected in the intertidal area of Malibu Beach (Los Angeles, USA) and were determined using taxonomic keys for the Californian Fauna [46,47] and afterwards this identification was checked by Leslie H. Harris (LACM Los Angeles). Specimens from San Diego were provided by Rick Rowe (San Diego) and have been collected in 25 m depth. European *Scoloplos *species have been all identified using the key from Hartmann-Schröder [6]. Voucher specimens for the Malibu clade, San Diego clade, type locality clade, and intertidal clade have been deposited in the collection "Vermes" of the Museum für Naturkunde der Humboldt-Universität zu Berlin (Germany) under the numbers 11213–11216. See Table 1 for sampling locations of European *Scoloplos*. DNA extraction was performed using the Qiagen DNeasy™ Tissue Kit (Qiagen, Germany) according to the manufacturer's instructions.

### mtDNA sequencing of *Scoloplos *cf. *armiger *individual SS14

To develop new genetic markers a 12 kb fragment including all coding genes was amplified from an individual of *Scoloplos *cf. *armiger*. The individual was collected subtidally near Sylt (Germany). In the first step small fractions of the *rrnL*, *cox1*, *cob*, and *nad4 *genes were amplified using conserved primers as described in Bleidorn et al. [35]. All products were purified with the Qiaquick PCR Purification Kit (Qiagen). Sequencing reactions were performed using the PCR primers with a dye terminator procedure and loaded on capillary automatic sequencer CEQ™ 8000 (Beckman Coulter, Fullerton CA, USA) according to the recommendations of the manufacturer.

In a second step the determined sequences were used to design three additional PCR primer pairs (Table 2) bridging the gaps between *rrnL*-*cox1*, *cox1*-*cob*, and *cob*-*nad4*. A long PCR approach using these primer pairs was performed using the Takara LA-Taq (MoBiTech). The 50 μl reaction volumes were set up as follows: 26.25 μl sterilized destilled water, 7 μl 10× reaction buffer, 7 μl MgCl-solution, 3.5 μl dNTP mix, 2 μl primer mix (10 μM each), 2 μl DNA template, 0.25 μl (1 u) Takara LA-Taq polymerase. A touchdown PCR approach was used for these fragments: 94°C for 3 min; 7 cycles with 94°C for 1 min, 63°C for 1 min (-0.5°C in every step), and 70°C for 8 min; 35 cycles with 94°C for 1 min, 60°C for 1 min 30 seconds, and 70°C for 8 min; final extension at 70°C for 10 min. PCR products were inspected under UV transillumination and a PCR purification of these four approximately 4 kb fragments was done using the PCR Gel extraction kit (Qiagen). Sequences were determined using direct sequencing from the ends of these fragments, then internally by primer walking.

### *cox3-trnQ-nad6 *amplification and sequencing

Using the mitochondrial genome data a primer pair spanning a ca. 600 bp region corresponding to the 3' end of *cox3*, *trnQ*, and most of the *nad6 *was designed (see Fig. 1 for priming sites on the genome, NAD6F: GGC TCW ACW TTC TTC GTA GCA CY, NAD6R: TTT TAC TGA RGC GAT TAR TGT TAG). All amplifications were carried out on an Mastercycler and Mastercycler gradient (Eppendorf). The PCR temperature reaction for this fragment was 94°C for 2 min; 34 cycles with 94°C for 30 seconds, 50°C for 45 seconds, and 70°C for 1 min; final extension at 70°C for 7 min.

All products were purified with the Qiaquick PCR Purification Kit (Qiagen). Sequencing reactions were performed with a dye terminator procedure and loaded on capillary automatic sequencer CEQ™ 8000 (Beckman Coulter, Fullerton CA, USA) according to the recommendations of the manufacturer. The trailing ends were trimmed, so that all sequences that were submitted to GenBank (for accession numbers see Table 1) are 528 bp in length.

### Gene annotation

Protein-coding genes and ribosomal RNA genes were identified by blasting on NCBI entrez databases and by comparing with other annelid mitochondrial genomes using DOGMA [48]. Boundaries of *nc *(the largest non-coding region) and the ribosomal genes could not be identified by sequence homology alone and were inferred from the boundaries of flanking genes. Transfer RNA genes were identified by their potential secondary structures using the tRNAscan-SE Search Server [49]. Transfer-RNA identity was specified by its anticodon sequence.

The sequence of the mitochondrial genome of *Scoloplos *cf. *armiger *individual SS14 has been submitted to GenBank (DQ517436).

### Phylogenetic analysis

Individuals possessing identical sequences were combined into a single operational taxonomic unit (OTU). Sequences were aligned with CLUSTAL W [50] using the default parameters for gap opening and gap penalty. Alignment of the protein coding regions was unambiguous, a few gap positions are only found within a non-coding region between *cox3 *and *trnQ *and within the transfer RNA. The alignment is available in treebase [51].

Phylogenetic analyses were carried out using PAUP*, version 4.0b10 [52] and MrBayes 3.0B4 [53]. According to the hypothesis of orbiniid phylogeny by Bleidorn [42] we used *Orbinia latreillii *as outgroup and this taxon served to root all trees. A chi-square test of homogeneity of base frequencies across taxa was used to estimate the frequency distribution of observed number of substitutional changes per character for each gene.

It is suggested that the Akaike Information Criterrion (AIC) is superior to the hierachical likelihood ratio test [54] and so we used this criterion for model selection as implemented in the program Modeltest 3.7 [55,56]. Average sequence distances were calculated using MEGA 2.1 [57].

Maximum likelihood analysis was performed under the likelihood settings suggested for the given dataset by the result of the modeltest using the heuristic search option with Tree Bisection Reconnection (TBR) branch swapping and 100 random sequence addition replicates. AIC indicates that GTR+I+Γ represents the optimal model in respect to the dataset (GTR = general time reversible, I = invariable sites, Γ = among-site rate variation modeled to fit a discrete gamma distribution).

Bootstrap values were determined from 1,000 replicates subject to full heuristic searches with simple addition sequence and NNI branch swapping to provide measures of relative clade support.

Bayesian analyses were conducted using MrBayes 3.0B4 [53]. All priors were set according to the chosen model (lset nst = 6 rates = invgamma; prset RevMatPr = dirichlet(1.0,1.0,1.0,1.0,1.0,1.0) StateFreqPr = dirichlet(1,1,1,1) ShapePr = uniform(0.05,50.0) PinVarPr = uniform(0.0,1.0)). Two times four Markov chains in parallel, three heated and one cold, were started from a random tree and all eight chains ran simultaneously for 1,000,000 generations, with trees being sampled every 500 generations for a total of 2,001 trees. After the likelihood of the trees of each chain converged, the first 101 trees were discarded as *burn in*. The majority-rule consensus tree containing the posterior probabilities of the phylogeny was determined from 1,900 trees.

An equally weighted maximum parsimony search was run with 1,000 random addition replicates, heuristic search option with TBR branch swapping, holding one tree per step, and keeping all most-parsimonious trees. Clade support was assessed with nonparametric bootstrap as implemented in PAUP* (heuristic search, 1,000 replicates, TBR branch swapping, and simple addition sequence).

## Abbreviations

AFLP, amplified fragment length polymorphism; *atp6 *and *8*, ATP synthase subunit 6 and 8; C, cytosine; *cox1-3*, cytochrome *c *oxidase subunits 1–3; *cob*, cytochrome *b *apoenzyme; *nad1-6 *and *4L*, NADH dehydrogenase subunits 1–6 and 4L; *nc*, noncoding region; *L*_1 _and *L*_2_, *trnL*(CUN) and *trnL*(UUR); RAPD, random amplified polymorphic DNA; *rrnS *and *rrnL*, small and large ribosomal RNA subunit; *S*_1 _and *S*_2_, *trnS*(AGN) and *trnS*(UCN); tRNA and *trn*, transfer RNA

## Authors' contributions

CB and IK planned the study. CB, IK, and SA carried out field samples. CB did most of the molecular genetic work, analysed the data and drafted the manuscript. IK and SA assisted in the molecular genetic work. TB supervised the work. All authors read and approved the final manuscript.
